# Bacterial Sepsis in Brazilian Children: A Trend Analysis from 1992 to 2006

**DOI:** 10.1371/journal.pone.0014817

**Published:** 2011-06-03

**Authors:** Cristina Malzoni Ferreira Mangia, Niranjan Kissoon, Otavio Augusto Branchini, Maria Cristina Andrade, Benjamin Israel Kopelman, Joe Carcillo

**Affiliations:** 1 Department of Pediatrics, Universidade Federal de São Paulo Escola Paulista de Medicina, São Paulo, Brazil; 2 Division of Critical Care, Department of Pediatrics, University of British Columbia, Child and Family Research Institute, Vancouver, Canada; 3 Hospital Emilio Ribas, São Paulo, Brazil; 4 Department of Anesthesiology and Critical Care Medicine, University of Pittsburgh School of Medicine, University of Pittsburgh, Pittsburgh, Pennsylvania, United States of America; The University of Hong Kong, Hong Kong

## Abstract

**Background:**

The objective of this study was to determine the epidemiology of hospitalized pediatric sepsis in Brazil (1992–2006) and to compare mortality caused by sepsis to that caused by other major childhood diseases.

**Methods and Findings:**

We performed a retrospective descriptive study of hospital admissions using a government database of all hospital affiliated with the Brazilian health system. We studied all hospitalizations in children from 28 days through 19 years with diagnosis of bacterial sepsis defined by the criteria of the International Classification of Diseases (ICD), (Appendix S1). Based on the data studied from 1992 through 2006, the pediatric hospital mortality rate was 1.23% and there were 556,073 pediatric admissions with bacterial sepsis with a mean mortality rate of 19.9%. There was a case reduction of 67% over.1992–2006 (p<0.001); however, the mortality rate remained unchanged (from 1992–1996, 20.5%; and from 2002–2006, 19.7%). Sepsis-hospital mortality rate was substantially higher than pneumonia (0.5%), HIV (3.3%), diarrhea (0.3%), undernutrition (2.3%), malaria (0.2%) and measles (0.7%). The human development index (HDI) and mortality rates (MR) by region were: North region 0.76 and 21.7%; Northeast region 0.72 and 27.1%; Central-West 0.81 and 23.5%; South region 0.83 and 12.2% and Southeast region 0.82 and 14.8%, respectively.

**Conclusions:**

We concluded that sepsis remains an important health problem in children in Brazil. The institution of universal primary care programs has been associated with substantially reduced sepsis incidence and therefore deaths**;** however, hospital mortality rates in children with sepsis remain unchanged. Implementation of additional health initiatives to reduce sepsis mortality in hospitalized patients could have great impact on childhood mortality rates in Brazil.

## Introduction

Sepsis remains the most important risk factor for mortality in infants and children in both industrialized and developing countries. Brazil has introduced universal access to primary care and immunizations to combat infectious disease in its vulnerable pediatric population. Recently, the World Federation of Pediatric Intensive Care and Critical Care Society launched the Global Pediatric Sepsis Initiative directed at improving outcomes from sepsis worldwide (www.wfpiccs.org). This initiative first calls for universal access to primary care and immunizations, as is already standard in Brazil. In addition, it stresses education and health care delivery organization to allow for early intravenous fluid resuscitation, antibiotic administration and goal directed therapies in the first hours of access to hospital care. This cost-effective approach has resulted in reduction in sepsis mortality rates from 20–22% to 1–2% in children in the United Kingdom and the Netherlands [1], [2].

The purpose of the present epidemiologic study is to:

1. Evaluate the changes in the admissions, costs and outcome of sepsis in hospitalized children from 1992 through 2006;
2. Determine the impact of Brazilian primary care on the incidence of Pediatric Sepsis;
3. Compare sepsis-related morbidity and mortality with morbidity and mortality secondary to others major childhood diseases;
4. Evaluate the potential for the goals set forth by the Global Sepsis Initiative to impact on mortality in Brazilian children.

## Methods

Our research is a retrospective descriptive study of hospital admissions for sepsis-related diagnosis among children and adolescents during fourteen-year period based on the records of the Hospital Information System of the Unified Health System (SUS) from Brazil [3]. The Brazilian healthcare system has provided universal access with the creation of the SUS in 1980. The hospitals included in the database in the last period were 5,100 hospitals and their regional distribution were 334 in north region, 1,539 in northeast, 1,613 in southeast, 988 in south and 626 in central-west region. The system ensures free medical care and accounts for approximately 80% of hospital admissions [3], [4].

The data were collected from the Hospital Information System of SUS (SIH-SUS) database that includes administrative information since 1984. SIH-SUS is an important font of health information because it has national coverage, a great number of variables (fifty), relatively easy for evaluation of data and short interval between registrations. The archives available are an administrative summary of all admissions in the hospitals affiliated with Brazilian SUS. The information collected is based on a Guideline of SIH-SUS. The admission diagnosis was established by an assistant physician of the hospital and this diagnosis was audited by a Committee of Review of Medical Records (CRMR), National Committee of Nosocomial Infection and National Committee of Diseases of Compulsory Notification.

During the period the admission diagnoses were considered the main diagnoses that lead to hospitalization based on the (International Classification of Diseases) ICD codes definition. Inconsistent data were removed from the system and returned to the original source for clarification. For death, we used the discharge diagnosis based in the death certificate available from National Mortality Information System (SIM).

The data from January, 1992 to December, 2006 were stored in two national databases (Tabnet and Tabwin) and the variables of interest were collected [3]. Furthermore, other causes of admissions and morbidity and mortality from the SIH-SUS were reviewed. We selected sepsis and the other major categories of admission for infections in children and compared the mortality rates for these groups.

An algorithm for extraction and manipulation of the data was built from the archives and implemented in an EXCEL spreadsheet, the data were stratified and coded before analysis, building files with the relevant information for all historical periods. These files were subsequently reviewed by two independent researchers by double keyboarding before analysis of epidemiological data. The first keyboarding and the second keyboarding were compared with original database in order to determine reliability of data.

In our study infection was defined as pathological process caused by the invasion of normally sterile tissue or fluid or body cavity by bacterial agents. The criteria for sepsis definition were considered as systemic inflammatory response syndrome (SIRS) plus infection. The criteria for SIRS in children were characterized by two or more of the following criteria: Temperature instability (hyperthermia >37.9°C or hypothermia <36°C), tachycardia, tachypnea and white blood cell count >2 SD above normal for age. In the database were included patients with bacterial sepsis confirmed by culture encoded by International Classification of Diseases (ICD 9 and 10) for bacterial sepsis but information about these specific bacterial agents is not available on the website studied.

Mortality by sepsis was defined according to ICD definition that stipulates one cause of death, which is considered to be the “disease or injury which initiated the train of morbid events leading directly to death” [3].

From January, 1992 to December, 1997 the classification used, was the 9th Revision of the Classification clinical modification (ICD-9-CM) and then the 10th Revision of the Classification (ICD-10) since January, 1998 [4]. Deaths were analyzed in the period and the mortality rate was calculated by ratio between the number of deaths and admissions by sepsis in the year considered, multiplied by 100.

In the present study, we used uniform criteria based on the ICD 9 and ICD 10 codes, because these codes for bacterial sepsis were constant from 1992 through 2006^.^ We used this criterion because it was only in 2003 that septic shock and severe sepsis were included as diagnoses in the International Classification of Diseases (3, Appendix S1).

The length of hospital stay (LOS) was defined in our study as an average of the length of stay.

Neonatal period is defined by Brazilian health system from 0 to 27 days. Children were defined as patients who were 19 years old or less excluding neonatal period (0 to 27 days of age). The age was stratified into five age groups: 28 days to under 1 year old, 1 to 4 years old, 5 to 9 years old, 10 to 14 years old and 15 to 19 years old as adopted by Brazilian Health Ministry [3]. Neonates (from 0 through 27 days) were excluded from this study because data from the neonatal period were housed in a different database and was not available for review.

The study was approved in September 19, 2008 under number 1170/08 by the Research Ethics Committee from Escola Paulista de Medicina, Universidade Federal de São Paulo, Brazil.

For comparison between Brazilian regions we used the Children Development Index (CDI) and Human Development Index (HDI) by Brazilian Regions based on the 2008 United Nations Development Program's Report [5], [6]. Annual data were stratified into three subperiods (from 1992 through 1996, 1997 through 2001 and 2002 through 2006) for assessment of temporal changes among cohorts. A simple linear regression analysis was used to examine linear trends among cohorts. Continuous data were analyzed using one way-ANOVA tests, and paired t-tests as appropriate. A p value of less than 0.05 was considered significant. The statistical analysis was performed by SPSS 15.0 software®, EXCEL 2002 (Microsoft®).

## Results

We identified 55,370,457 children hospital admissions during the study period. Table 1 reports the ICD codes related to causes of hospitalization Brazilian children or 55,351,894 hospital admissions. By ICD codes, infectious and parasitic diseases, diseases of respiratory system, diseases of the digestive system, complications of pregnancy, childbirth, puerperium and injury represent approximately 75% of all hospital admissions (Table 1). The age distribution by admission diagnosis showed that proportion of admissions were highest in children under 1 year (11,804,432 admissions) and among 1 to 4 years (14,214,455 admissions) considering age-group risk specific. The top illness-related causes of hospitalization under 1 year old corresponding to 87% of all admissions are presented in Table 2.

**Table 1:** Hospital admissions by main ICD codes according to subperiods.

From 28 days to 19 years	1992–1996			1997–2001			2002–2006		
ICD Chapters	Admission^*^	%^†^	Rate^‡^	Admission^*^	%^†^	Rate^‡^	Admission^*^	%^†^	Rate^‡^
Infectious and parasitic diseases	3,753,957	18.19	2.21	2,573,141	14.17	1.88	2,607,905	15.75	1.2
Neoplasms	271,371	1.31	3	217,222	1.20	3.26	360,665	2.18	2.3
Diseases of the blood and blood-forming organs	709,582	3.44	1.98	117,779	0.65	1.53	122,804	0.74	1.16
Endocrine, nutritional and metabolic	709,582	3.44	1.98	424,552	2.34	1.425	365,243	2.21	0.9
Mental disorders	920,55	0.45	0.13	88,749	0.49	0.105	74,396	0.45	0.1
Diseases of the nervous system	523,230	2.53	2.66	301,272	1.66	3.065	244,693	1.48	2.75
Diseases of circulatory system	232,587	1.13	5.16	153,423	0.85	5.61	120,393	0.73	5.56
Diseases of respiratory system	6,108,257	29.59	0.85	5,055,189	27.85	0.71	4,373,589	26.42	0.61
Diseases of the digestive system	959,737	4.65	0.79	1,097,455	6.05	0.72	989,780	5.98	0.62
Diseases of the skin and subcutaneous tissue	202,991	0.98	0.21	198,766	1.09	0.17	217,989	1.32	0.15
Diseases of the musculoskeletal system	316,437	1.53	0.21	244,111	1.34	0.285	224,594	1.36	0.23
Diseases of the genitourinary system	947,184	4.59	0.28	715,307	3.94	0.26	670,407	4.05	0.23
Complications of pregnancy, childbirth, puerperium	3,853,255	18.67	0.03	3,918,000	21.58	0.02	3,335,771	20.15	0.02
Certain conditions originating in the perinatal period	585,746	2.84	7.5	1,095,923	6.04	7.76	1,015,772	6.14	6.51
Congenital anomalies	243,148	1.18	3.43	284,510	1.57	3.295	312,788	1.89	3.19
Injury and poisoning	1,363,894	6.61	1.11	1,007,775	5.55	1.14	108,6491	6.56	1.16
External				85,297	0.47	1.37	8,278	0.05	0.93
Total	20,641,971	100.00	1.32	18,154,477	100.00	1.255	16,555,446	100.00	1.14

**Table 2:** Illness-related hospitalizations by primary diagnosis admission under 1 year from 2002 to 2006.

Rank	Principal Diagnosis at Discharge	Number	Percent*	Mortality rate
1	Pneumonia	708948	21.22	0.95
2	Other disorders originating in the perinatal period	271743	8.13	9.74
3	Other bacterial intestinal infections	257086	7.70	1.18
4	Disorders related to short gestation and low birth weight	251837	7.54	10.94
5	Diarrhea and gastroenteritis of presumed infectious origin	236232	7.07	0.48
6	Other disorders originating in the perinatal period	228409	6.84	1
7	Asthma	160569	4.81	0.17
8	Acute bronchiolitis and bronchitis	119753	3.58	0.26
9	Other diseases of the respiratory system	84326	2.52	8.9
10	Influenza [flu]	75528	2.26	0.65
11	Other infections specific to the perinatal period	68278	2.04	2.18
12	Sepsis	60110	1.80	21.92
13	Volume depletion	53218	1.59	1
14	Other diseases of intestines and peritoneum	52632	1.58	1.51
15	Acute laryngitis and tracheitis	43175	1.29	0.1
16	Intrauterine hypoxia and birth asphyxia	39101	1.17	9.62
17	Other specified congenital infectious and parasitic diseases	37533	1.12	10
18	Inguinal hérnia	33493	1.00	0.15
19	Congenital malformations of the circulatory system	32465	0.97	16.71
20	Malnutrition	28082	0.84	4.11
21	Renal tubulo-interstitial diseases	26370	0.79	0.16
22	Congenital syphilis	19057	0.57	0.34
23	Epilepsy	18395	0.55	0.94
24	Iron deficiency anaemia and other nutritional anaemias	13362	0.40	1.62
25	Trauma Brain Injury	10678	0.32	2.76
	Total	2930380	87.72*	4.29

The total admissions in the period from 2002 to 2006 were 3,340,575.

The diagnoses listed were the top 25 most common illness-related hospitalizations by primary admission diagnosis.

Sepsis-related causes of hospitalization are presented in Table 3. During the entire period infectious and parasitic diseases represented 16% of admissions while sepsis 1%. In the same period there were 610,787 deaths among children under 19 years. Death in children under 1 year old contributed to most deaths (74%) among all diseases and by sepsis as well (13.6%). Among overall sepsis mortality (106,989 deaths), children under 5 year old was most common with 91% or (99,168 deaths). The total number of cases of sepsis in the first period there were 284,432 cases and admission rate 11.2 cases per 1000 admissions. In the last period the total number of cases was 101,570 cases with admission rate of 6 cases per 1000 admissions, or case reduction of 64% (p<0.001).

**Table 3:** Characteristics of patients with sepsis, according to subperiod.

	1992–1996	1997–2001	2002–2006
Hospital admissions	20,642,002	18,173,097	16,555,358
LOS* (mean±SD)	4.8±1.6	4.5±0.1	4.5±1.2
Hospital days	100,106,163	82,544,797	73,862,025
Mortality rate	1.3%	1.3%	1.1%
Costs/patient (USD)	103.79±3.38	141.37±27.38	216.26±47.40
Sepsis admissions(M/F)	282,137(55%/45%)	172,366(55%/45%)	101,570(55%/45%)
1 mo[–1 year(M/F)	182,819(56%/44%)	103,600(56%/44%)	60,110(56%/44%)
1year[–5 year(M/F)	57,375(53%/47%)	37,443(53%/47%)	20,473(53%/47%)
≥5 year(M–F)	41,943(54%/46%)	31,422(54%/46%)	20,987(54%/46%)
Hospital days	2,851,723	1,850,631	1,279,304
1 mo [–1 year	1,901,016	1,168,659	807,447
1year [–5 year	553,332	370,456	232,534
≥5 year	397,375	311,516	239,323
LOS (mean±SD)	10.1±0.3	10.8±0.6	12.6±0.6
1 mo [–1 year	10.4±0.3	11.5±0.8	13.4±0.6
1year [–5 year	9.6±0.3	9.9±0.2	11.4±0.5
≥5 years	9.5±0.4	9.9±0.3	11.4±0.6
Mortality	57,932	33,946	20,048
1 mo [–1 year	45,372	24,655	13,178
1year [–5 year	7,677	4,971	3,315
≥5 years	4,883	4,320	3,555
Mortality rate	20.53	19.37	19.74
1 mo [–1 year	24.82	23.54	21.92
1year [–5 year	13.38	12.6	16.19
≥5 years	11.64	13.65	16.94

*Length of Stay (LOS).

**Figure 1:**
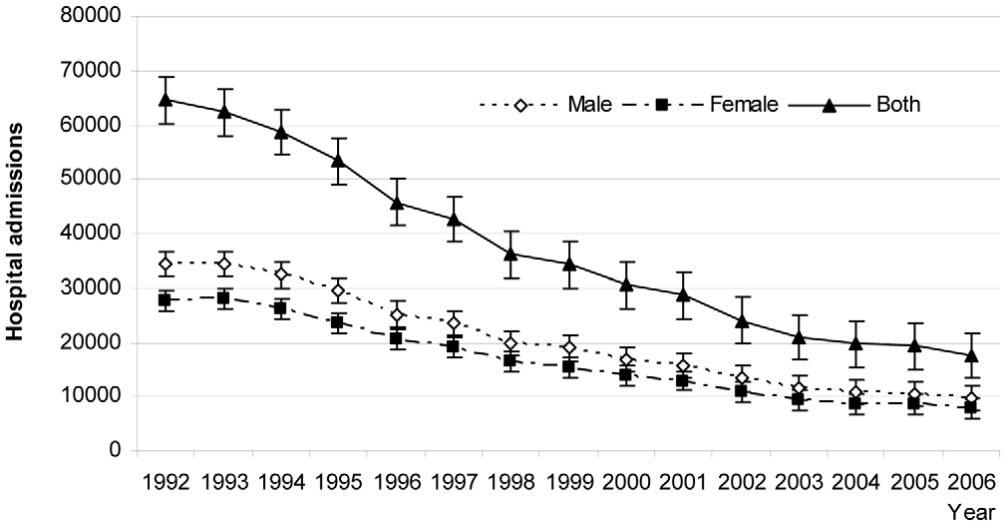
Trends in hospital admissions over 1992 – 2006.

The total number of cases was highest in children between 1 month and 1 year from the first to last period (182,809 cases to 60,100 cases), and there was a case reduction of 67% over these periods. The rate of admissions for sepsis decreased progressively from children to adolescents (Table 3; p<0.001, Figure 1).

The admissions was highest in children between 1 month to 1 year old from the first to last cohort (182,809 cases to 60,100 cases) with case reduction of the 67% over these periods. The admissions decreased progressively from children to adolescents (Table 3; p<0.001).

Boys were most susceptible to sepsis from the first to last cohort (Table 3). Boys also had a significantly higher sepsis-related hospitalization than girls (mean difference 3853.67 [95% confidence interval for difference 2897.16 to 4810.17; p = .000]) when considering all age-groups.

The mortality decreased 64% from the first to the last cohort over 14-year period. The mortality was 57,932 (mortality rate; 20.4% by 100 admissions); 33,946 (mortality rate, 20.5%) and 20,048 deaths (mortality rate, 19.7%) before discharge from the first period to the last period (Table 4).

**Table 4:** Hospital admissions for sepsis by age group.

Years	1 mo|----1 y	1 |----4 y	5|----9 y	10|----14 y	15|----19 y	Total
1992	41,058	12,325	3,599	2,486	2,747	62,215
1993	40,924	12,513	3,482	2,655	2,683	62,257
1994	38,159	11,594	3,602	2,625	2,687	58,667
1995	33,858	10,922	3,518	2,386	2,628	53,312
1996	28,820	10,021	2,920	2,015	1,910	45,686
1997	26,029	9,788	2,905	1,887	2,043	42,652
1998	21,250	8,542	2,435	1,799	2,169	36,195
1999	20,227	7,556	2,407	1,875	2,261	34,326
2001	17,304	5,622	2,112	1,572	2,092	28,702
2002	14,320	4,910	1,771	1,295	1,698	23,994
2003	12,260	4,447	1,633	1,158	1,384	20,882
2004	11,574	4,061	1,488	1,216	1,372	19,711
2005	11,465	3,647	1,712	1,123	1,333	19,280
2006	10,465	3,355	1,469	1,086	1,213	17,588

**Figure 2:**
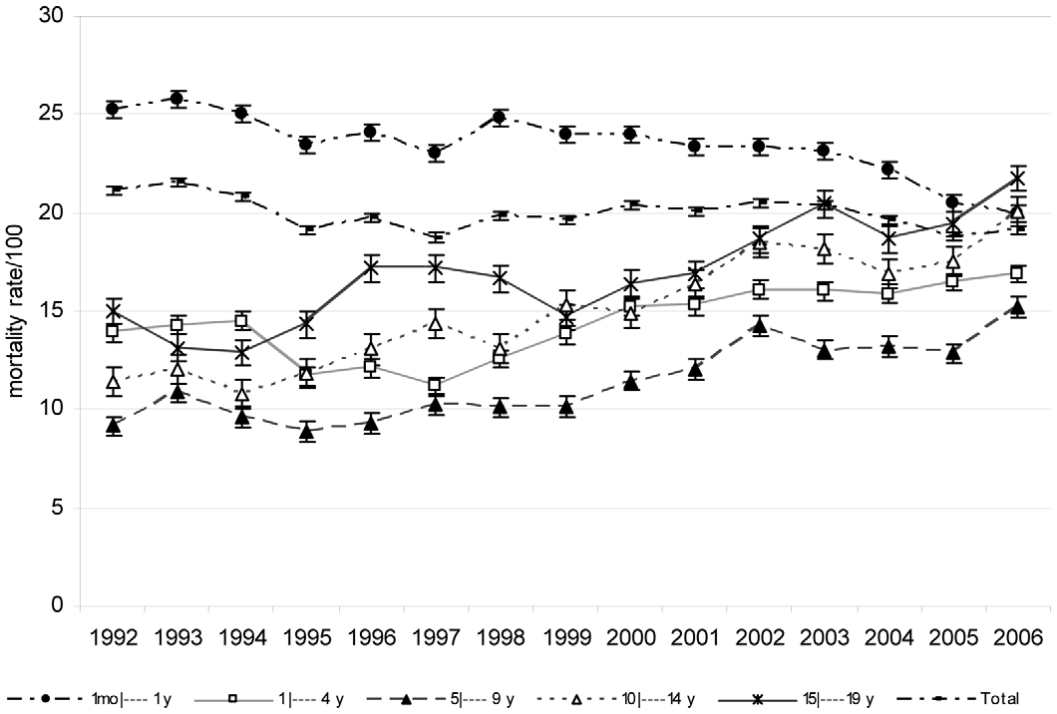
Trends in hospital mortality over 1992 – 2006.

Mortality rates from 1992 to 2006 remained static over the 14-year period, averaging 20.5% in the first cohort and 19.7% in the last cohort (p = .300) (Figure 2).

**Figure 3:**
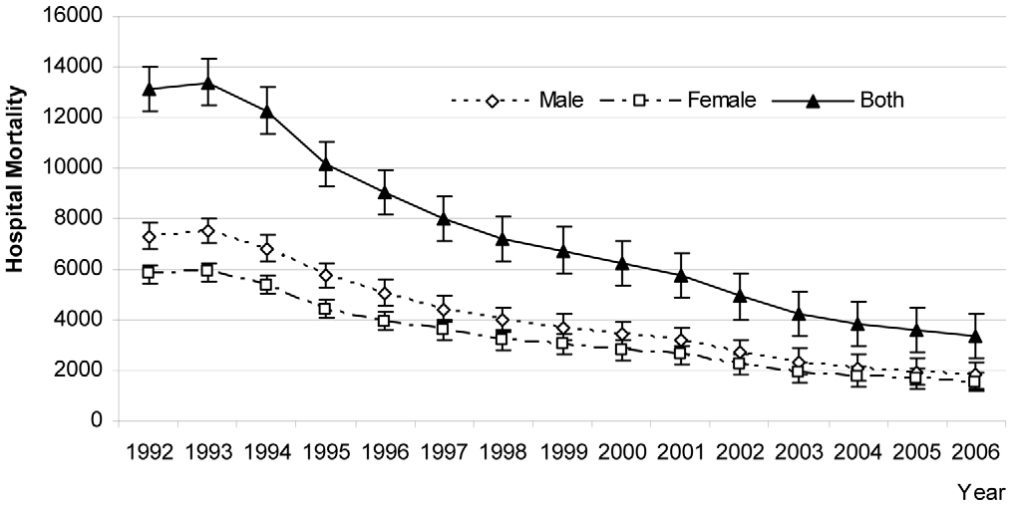
Trends in hospital mortality rates. Legend:---◊--- Male ---▪---Female ---▴--- Both.

**Figure 4:**
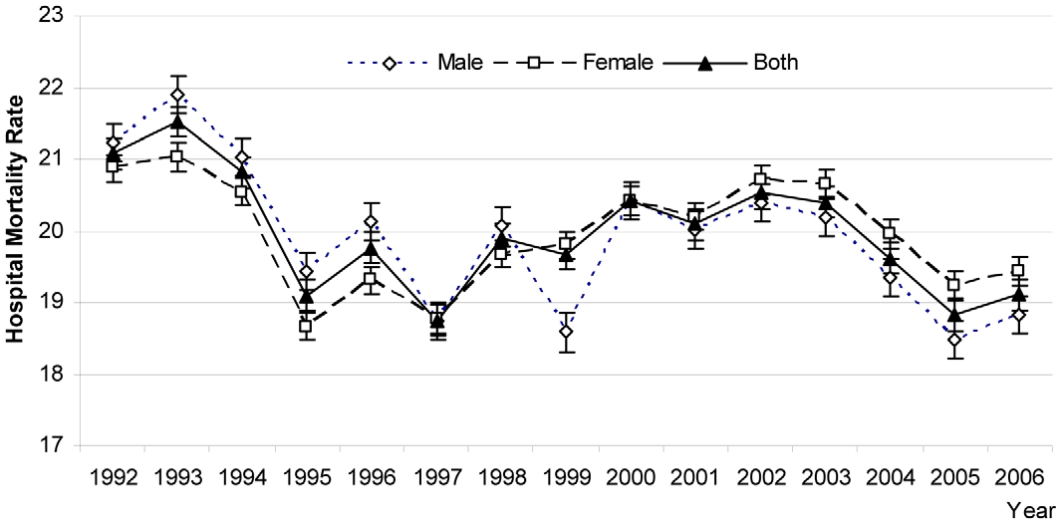
Hospital Sepsis mortality rate by age and group categories, overall mortality unchanged over the study period 1992–2006. Legend:---•---1 mo ---□---1---4 y ---▴--- 5---9 y ---Δ--- 10---14 y ---*--- 5---19 y ---•---Total.

By age group, the mortality rate among children under 1 year old declined over the 14-year period (p = 0.023) but increased among children 1 to 4 years (p<.05) and among children above 5 years (p <0.01) for the entire study period (Figure 2, Table 4). The hospital mortality differed significantly between boys and girls, mean difference, 818.20 (95% confidence interval for difference 559.45 to 1076.95; p = .000) among cohorts (Figure 3). In addition, the mortality rate in girls was higher than boys under 5 years in the last cohort (p<.05) (Figure 4).

The hospital admissions by bacterial sepsis were different among Brazilian regions. In 2006, there were 2,155 admissions (467 deaths) in North region; 4,769 (1292 deaths) in Northeast; 6,295 (931 deaths) in Southeast; 3,119 (380 deaths) in South and 1,250 (293 deaths) in Central-West region (p = 0.000) (Table 5).

**Table 5:** Mortality rate of sepsis cases during hospitalization (%) by age group.

Years	1 mo|----1 y	1 |---- 4 y	5|---- 9 y	10|----14 y	15|----19 y	Total
1992	25.26	13.93	9.14	11.42	15.00	21.08
1993	25.80	14.28	10.83	12.05	13.12	21.52
1994	25.05	14.52	9.61	10.78	12.88	20.82
1995	23.43	11.68	8.87	11.82	14.35	19.10
1996	24.12	12.10	9.32	13.05	17.17	19.76
1997	22.99	11,17	10.26	14.41	17.18	18.75
1998	24.86	12,57	10.10	13.06	16.64	19.89
1999	23.97	13.82	10.14	15.31	14.68	19.68
2001	23.36	15.28	12.07	16.34	16.87	20.09
2002	23.34	16.07	14.29	18.53	18.67	20.53
2003	23.17	16.03	13.04	18.13	20.45	20.40
2004	22.16	15.86	13.24	16.86	18.66	19.62
2005	20.51	16.48	12.85	17.54	19.43	18.82
2006	19.96	16.90	15.18	20.07	21.76	19.11

In regional terms and considering the Child Development Index (CDI) and Human Development Index (HDI) by regions, the mortality rates in the North region which has a medium Child Development Index (CDI  = 0.56; HDI  = 0.76) was 21.7%, Northeast region (CDI  = 0.56; HDI = 0.72) was 27.1% and Central-West (CDI  = 0.70; HDI = 0.81) was 23.5%. These were two fold higher than the South region (CDI  = 0.73; HDI = 0.83) (mortality rate 12.2%). The North region, had a mortality rate similar to the Brazilian average (19.1%; CDI = 0.67; HDI = 0.79). In the Southeast region (CDI = 0.75; HDI = 0.82), which is industrialized and considered a less vulnerable region of Brazil, the mortality rate was 14.8%. However, in São Paulo State one the most industrialized and developed states of Brazil with CDI classified as high (CDI  = 0.8; HDI = 0.83), the mortality rate by sepsis was two fold higher than the South region (20,8% versus 12.1%, respectively). This rate is comparable to other less-favoured regions such as the North (21.7%), Central-West (23.4%) and Northeast region (27.1%) and reflects intra-regional disparities. The mortality rate was not significant considering CDI (p = 0.132) and but was based in the Human Development Index (HDI) (p = 0.008) by regions.

The incidence of the childhood diseases including sepsis and other infections with a high potential of morbidity and mortality were studied over the 14-year period (Table 6). In 2002–2006, the mortality rate of diarrhea, measles, pneumonia and malaria were 0.3%; 0.7%; 0.5% and 0.2%. The incidence of diarrhea, measles, pneumonia, and malaria declined 61%; 94.2%; 58% and 77%, respectively over the three time periods. The mortality rate for bacterial sepsis was increased 20 fold or more when compared with every other condition except meningococcal disease (Table 6).

**Table 6:** Mortality rate (%) in 2006 for sepsis by Brazilian regions and age groups.

	North*	Northeast#	Southeast§	South†	Central West‡	São Paulo 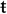 [Table-fn nt109]	Total¤
1 mo[----1 y	25.5	30.2	13.3	10.7	27.1	17.1	20
1 |---- 4 y	18.0	19,4	14.3	16.1	19.9	22.1	17
5 |---- 9 y	16.5	19.3	14.3	10.3	15.6	22.1	15.1
10 |---- 14 y	16.2	23.9	21.4	15.2	17.9	31.1	20.1
15 |---- 19 y	15.3	31.7	23.1	14.9	16.8	31.7	21.8
TOTAL	21.7	27.1	14.8	12.1	23.4	20.7	19.11

*CDI (children development index) of North region  = 0.56.

#CDI of Northeast region  = 0.56.

§ CDI of Southeast region  = 0.75.

† CDI of South region  = 0.73.

‡ CDI of Central West region = 0.70.

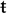
CDI of São Paulo State  = 0.80.

¤CDI of Brazil  = 0.67.

## Discussion

Our study is the first observational, longitudinal, population-based analysis of temporal trends using a national database examining sepsis in Brazil. This analysis demonstrates that although sepsis is a grave public health issue, considering its high mortality, the number of children sepsis-related hospitalization decreased by 64% from 1992 through 2006 in Brazilian children. This decrease might be explained in part by the implementation of the Millennium Development Goals (MDG) with the objective to reduce child mortality since 1990 [7], [8]. In this regard, in 2006 the birth rate decreased to 2.0 children per woman and the child mortality rate (under 5 years) was 24.9 per thousand. In addition, there was an increase in the number of national programs to improve nutritional status [4]. A recent national survey estimated the proportion of malnutrition in children with low body-weight by age under 1 year was 3.6% and under 2 years was 7.7% in 2004 [4]. In the period studied, there was a decrease in the incidence and mortality from infectious diseases, associated with implementation of public health programs directed to providing increased access to vaccination (full free vaccination coverage programs), and increased use of oral rehydration and reduction in the incidence of diarrhea (mainly in the post-neonatal period). In addition, from 1992 through 2006, there were improvements in the sanitation and trash collection services as well as water treatment. The population coverage indicators in 2005 (which is the most recent year with data available) for immunization, sanitation, trash collection services and water treatment included 93%, 67%, 84% and 81% of the Brazilian population, respectively [3].

These excellent outcomes were associated with the implementation of the Brazilian Millennium Development Goals and primary care initiatives; however, not unexpectedly, this initiative had little effect on hospital based mortality once sepsis occurred. A study examining U.S. pediatric sepsis demonstrated a hospital mortality rate of 9.5 % in the 1990s compared to a more recent study which found a hospital mortality rate of 4.6% compared to 19% in our Brazilian cohort [9], [10].

High in-hospital mortality rates observed among cohorts can be a reflection of social inequities such as late presentation, the quality of care rendered, lack of adherence to sepsis clinical practice guidelines, as well as lack of access to preventive care, and early diagnostic and screening services. Other possibilities could include differences of patient mix such as emergent pathology that includes increased survival in cancer patients, transplant and other comorbidities which are important reasons for increase of sepsis episodes and mortality [11], [12]. However, we did not observe this association in our study and hence the reason for the maintenance of high mortality rates remains unclear. A limitation of this research was the cross-tabulation between primary cause of hospitalization and comorbidities was not possible in SIH-SUS database. The primary cause is reported as the main admission diagnosis that which led to patient hospitalization, and is adopted by ICD 9 and 10 definitions. For admission, there is a clinical reporting form used for all hospitals which include primary, secondary causes and comorbidities, meanwhile cross-tabulation between sepsis and comorbidities is a limitation of this database.

St Mary's Hospital in the United Kingdom first reported a reduction in hospitalized pediatric sepsis mortality from 22% to 2 % when education, emergency department care and a hospital transport system was introduced to improve outcomes in sepsis. The key to success was early fluid resuscitation and antibiotic administration by intravenous catheters followed by goal directed therapy usually consisting of a peripheral and or centrally administered inotropic agent. Sophia Children's Hospital in Rotterdam more recently reported a reduction in mortality from 20 % to 1% with this approach [1], [2].This initiative has not yet been implemented in Brazil and could explain in part the lack of improvement in mortality rates observed.

Sepsis morbidity in our cohort was high in 2002–2006 and the mean length of stay was three-fold higher than the mean of all pediatric admissions. However the mean hospital LOS in our population of 12.6 days was extremely low when compared with a study of American children (24 days) [8], [9], [10]. By regions, the sepsis-related mortality rate was different mainly in São Paulo State. São Paulo has highest population coverage indicators of country for immunization, sanitation, trash collection services and water treatment. In addition, the population resident in São Paulo has highest gross internal product compared to others regions. Recent data related to others Brazilian metropolitan areas has showed reduction of poverty rate from 32,9% to 24,1% between 2002 to 2008 [3].

A recent study described causes of hospitalization, in the public health system, of children from zero to nine years in São Paulo city over a period from 2002 through 2006, shows an increase in the number of hospital admissions while in the rest of Brazil the trend was a decrease. Several factors were hypothesized to explain this fact as individual predisposition, enabling factors (factors that promote or discourage the use of health system). However, these factors alone or in association cannot explain the increases seen [11].

We found an important difference in outcomes when comparing sepsis with other major causes of death in children including pneumonia, diarrhea, malaria, measles, undernutrition and HIV. Indeed, Brazilian mortality rates from sepsis and meningococcal disease were higher than all other infectious diseases studied. Our findings confirm previous studies where bacterial sepsis was associated with high mortality, and consumed more healthcare resources as compared with others diseases [9]. Our study findings suggest that a critical review to determine the reason for the high mortality seen in bacterial sepsis and meningococcal disease and efforts to address same is warranted.

Our study has some limitations to consider. An important point that could be considered was that the definition of sepsis was non-uniform because the final diagnosis was established by the assistant physician and may be prone to bias. This aspect is very important because while in adults the sepsis definition is relatively easy, the same is not true for children and this has led to several definitions depending on age grouping [12], [13], [14], [15]. In addition, as distinct from adults, children frequently present in the hospital with the inflammatory triad of fever, tachycardia, and vasodilation and very few of these children have sepsis [16], [17], [18]. So, early and accurate diagnosis of sepsis in children is not easy and is clearly related to medical ability, perception and physician knowledge about sepsis related physiological abnormalities in different age-groups [15].

Sepsis-related LOS was presented in-year hospital days in the SIH-SUS. We used the average time of hospitalization for sepsis, therefore we were unable to determine the length of admissions and evaluate any relationship between sepsis and chronic conditions in sepsis-related admissions. As well, the SIH-SUS database is based in clinical criteria of sepsis-related hospitalization admission this fact represents a problem concerning to the reliability of the diagnosis, where other specified septicemia (A41.8), septicemia, unspecified, septic shock (A41.9) may be classified as sepsis (Appendix S1). Another limitation is that database does not include important aspects of sepsis such as details of the microbiologic agents for diagnosis of bacterial sepsis.

An important aspect concern relates to intensive care admissions because the implementation of pediatric intensive care units (PICU) on a large scale in Brazil is recent [19].There are no national statistics about quality of care indicators in the public and private PICU in Brazil [3].Therefore the specific data were scarce or non existent during the period of this study [19]. In addition, there was no information about transportation, hospital and PICU access by population. Indeed, the universal public emergency transport system with basic life support and/or advanced life support was implemented in our country only in 2003 [20].

Our research focused in the fourteen-year period because there were a number of initiatives, such as WHO Millennium Development Goals and consensus conferences that clarified the understanding and prevention and treatment of sepsis and sepsis in children [13], [14], [21]–[30].These initiatives address prevention, early recognition and use of goal-directed therapies. But few Brazilian pediatric centers adhere fully to these guidelines [31].Many reasons exist for lack of implementation of these recommendations in practice, but organizational issues may account for low adherence to guidelines in the country [31], [32]. Another possibility is that the clear, objective and easy information about disease is not available to those taking care of most children with sepsis [23], [24]. The implementation of educational and interventions continuing programs for adequately trained staff in the hospital care of children as well as policy-relevant research agendas, donor funding partnerships and development of prehospital and transports programs could potentially reduce sepsis incidence and mortality [31].

In conclusion, the creation of international initiatives with full participation of developing countries to better understand the sepsis problem globally as well as the regional inequities which hinder measures to combat diseases is needed.

## Supporting Information

Appendix S1 International Classifications of Diseases (ICD) for Sepsis.
(0.08 MB DOC)
