# Supplementary material for: Bacterial Sepsis in Brazilian Children: A Trend Analysis from 1992 to 2006
Source: PLoS One. 2011 Jun 3;6(6):e14817. doi: 10.1371/journal.pone.0014817 (PMC3108592; doi:10.1371/journal.pone.0014817)
Supplement: Appendix S1 — International Classifications of Diseases (ICD) for Sepsis. (0.08 MB DOC) [file pone.0014817.s001.doc]

ICD 9 Codes for Sepsis from 1992 to 1997

| 038 | Septicemia   - Use additional code for systemic inflammatory response syndrome (SIRS)   Excludes:   - Bacteremia - Septicemia (sepsis) of newborn | 995.91-995.92  790.7  771.81 |
| --- | --- | --- |
| 038.0 | Streptococcal septicemia |  |
| 038.1 | Staphylococcal septicemia |  |
| 038.10 | Staphylococcal septicemia, unspecified |  |
| 038.11 | Methicillin susceptible Staphylococcus aureus septicemia  MSSA septicemia  Staphylococcus aureus septicemia NOS |  |
| 038.12 | Methicillin resistant Staphylococcus aureus septicemia |  |
| 038.19 | Other staphylococcal septicemia |  |
| 038.2 | Pneumococcal septicemia [Streptococcus pneumoniae septicemia] |  |
| 038.3 | Septicemia due to anaerobes  Septicemia due to bacteroides  Excludes:   - Gas gangrene - Due to anaerobic streptococci | 040.0  038.0 |
| 038.4 | Septicemia due to other gram-negative organisms |  |
| 038.40 | Gram-negative organism  Unspecified Gram-negative septicemia NOS |  |
| 038.41 | Hemophilus influenzae [H. influenzae] |  |
| 038.42 | Escherichia coli [E. coli] |  |
| 038.43 | Pseudomonas |  |
| 038.44 | Serratia |  |
| 038.49 | Other |  |
| 038.8 | Other specified septicemias  Excludes:   - Septicemia due to:   - Anthrax   - Gonococcal   - Herpetic   - Meningococcal   - Septicemic plague | 022.3  098.89  054.5  036.2  020.2 |
| 038.9 | Unspecified septicemia  Septicemia NOS  Excludes:   - Bacteremia NOS | 790.7 |

ICD 10 Codes for Sepsis (A40 and A41) from 1998 to 2006

| A40 | Streptococcal sepsis   - Use additional code to identify resistance to antimicrobial and antineoplastic drugs   Excludes:   - During labour - Following   - Abortion or ectopic or molar pregnancy   - Immunization   - Infusion, transfusion or therapeutic injection   - Neonatal   - Postprocedural   - Puerperal   - Sepsis due to Enterococcus | U82-U85  075.3  O03-O07, O08.0  T88.0  T80.2  P36.0-P36.1  T81.4  O85  A41.80 |
| --- | --- | --- |
| A40.0 | Sepsis due to streptococcus, group A |  |
| A40.1 | Sepsis due to streptococcus, group B |  |
| A40.2 | Sepsis due to streptococcus, group D |  |
| A40.3 | Sepsis due to streptococcus pneumoniae  Pneumococcal sepsis |  |
| A40.8 | Other streptococcal sepsis |  |
| A40.9 | Streptococcal sepsis, unspecified |  |
| A41  A41  *continued* | Other sepsis   - Use additional code to identify resistance to antimicrobial and antineoplastic drugs   Excludes:   - During labour - Following:   - Abortion or ectopic or molar pregnancy   - Immunization   - Infusion, transfusion or therapeutic injection   - Sepsis due to or in:     - Actinomycotic     - Anthrax     - Candidal     - Erysipelothrix     - Extraintestinal yersiniosis     - Gonococcal     - Herpesviral     - Listerial     - Meningococcal     - Neonatal     - Postprocedural     - Puerperal     - Streptococcal     - Tularaemia   Excludes:   - Septic:   - Melioidoses   - Plague   - Toxic shock syndrome | U82-U85  075.3  O03-O07, O08.0  T88.0  T80.2  A42.7  A22.7  B37.7  A26.7  A28.2  A54.8  B00.7  A32.7  A39.2-A39.4  P36.-  T81.4  O85  A40.-  A21.7  A24.1  A20.7  A48.3 |
| A41.0 | Sepsis due to Staphylococcus aureus |  |
| A41.1 | Sepsis due to other specified staphylococcus  Sepsis due to coagulase-negative staphylococcus |  |
| A41.2 | Sepsis due to unspecified staphylococcus |  |
| A41.3 | Sepsis due to Haemophilus influenzae |  |
| A41.4 | Sepsis due to anaerobes  Excludes:   - Gas gangrene | A48.0 |
| A41.5 | Sepsis due to other gram-negative organisms |  |
| A41.50 | Sepsis due to Escherichia coli [E coli] |  |
| A41.51 | Sepsis due to Pseudomonas  Sepsis due to pseudomonas aeroginosa |  |
| A41.52 | Sepsis due to Serratia |  |
| A41.58 | Sepsis due to other gram-negative organisms  Gram-negative sepsis NOS |  |
| A41.8 | Other specified sepsis |  |
| A41.80 | Sepsis due to enterococcus   - Excludes sepsis due to Streptococcus D | A40.2 |
| A41.88 | Other specified sepsis |  |
| A41.9 | Sepsis, unspecified  Septicemia   - Use additional code to identify septic shock | R57.2 |
| A41.9 | Bacteraemia NOS |  |
